# Bothersome Pelvic Floor Dysfunction and Quality of Life During Pregnancy and Postpartum in Primiparous Women

**DOI:** 10.1007/s00192-024-06038-1

**Published:** 2025-01-20

**Authors:** Sofia Nestor, Jan Brynhildsen, Ayako Hiyoshi, Markus Harry Jansson

**Affiliations:** 1 Department of Obstetrics and Gynecology, Faculty of Medicine and Health, Örebro University, Örebro, Sweden; 2 School of Medical Sciences, Faculty of Health and Medicine, Örebro University, Örebro, Sweden; 3 Clinical Epidemiology and Biostatistics, Faculty of Medicine and Health, Örebro University, Örebro, Sweden; 4 Department of Clinical Sciences, Danderyd Hospital, Karolinska Institutet, Stockholm, Sweden; 5 Department of Obstetrics and Gynecology, Örebro University Hospital, Region Örebro County, PO Box 1613, 701 16 Örebro, Sweden

**Keywords:** Urinary incontinence, Childbirth, Health-related quality of life, Pelvic floor disorder, Postpartum

## Abstract

**Introduction and Hypothesis:**

This is a prospective cohort study based on the hypothesis that pregnancy and childbirth are associated with the occurrence of bothersome pelvic floor dysfunction (PFD), which impairs health-related quality of life (HRQoL).

**Methods:**

Primiparous women completed a questionnaire including questions about bothersome PFD and HRQoL in early pregnancy, late pregnancy, 8 weeks postpartum, and 12 months postpartum. HRQoL was measured using the Incontinence Impact Questionnaire, Short Form (IIQ-7). The association between bothersome urinary incontinence (UI) 1 year postpartum and maternal and delivery characteristics was examined using generalized linear models.

**Results:**

The study sample comprised 851 women. The prevalence of bothersome UI increased from 0.7% in early pregnancy to 8.1% at 1 year postpartum. At 1 year postpartum, 22.8% of the women reported PFD with impairment in HRQoL, with a median IIQ-7 score of 14.3. Bothersome UI in late pregnancy (adjusted RR 4.51, 95% CI 1.43–14.26) and 8 weeks postpartum (adjusted RR 10.17, 95% CI 5.45–18.98) were associated with bothersome UI 1 year postpartum.

**Conclusions:**

Most women were not bothered by UI during pregnancy and up to 1 year postpartum and did not report PFD with impairment in HRQoL. Most women who reported PFD with impairment in HRQoL had low IIQ-7 scores, but a few women reported substantial restriction in lifestyle. Bothersome UI in late pregnancy and 8 weeks postpartum was predictive of bothersome UI 1 year postpartum. This indicates the importance of the early identification of these women to provide appropriate counseling and treatment.

## Introduction

Pelvic floor dysfunction (PFD) is common in adult women [1] and has a great impact on health-related quality of life (HRQoL), including physical, psychological, and social well-being [2]. PFD comprises a wide variety of conditions, including urinary incontinence (UI), fecal incontinence (FI), and pelvic organ prolapse (POP) [3]. UI in women is divided into stress urinary incontinence (SUI), urgency urinary incontinence (UUI), or a combination of these, called mixed urinary incontinence (MUI) [3]. POP is the descent of any of the vaginal walls, the uterus, or the apex of the vagina. Sensation of tissue protrusion from the vagina, known as vaginal bulging, is the most specific symptom of POP [4]. It appears that both pregnancy per se and vaginal delivery may contribute to PFD, with UI being the most common early manifestation [5]. The extent to which pregnancy and vaginal delivery contribute to different types of PFD appears to differ. The degree of inconvenience of UI and the impact on HRQoL rather than symptom severity appear to drive women to seek treatment [6].

Few studies have evaluated the bother of UI or the impact of UI on HRQoL in women after childbirth [5, 7–9]. Van Brummen et al. studied bothersome lower urinary tract symptoms (LUTS) and found that bothersome SUI in early pregnancy and higher maternal age were predictive of bothersome SUI at 1 year postpartum. However, they concluded that, overall, most women were not bothered by their LUTS after their first delivery [8].

The role of delivery mode in the development of bothersome UI and the delivery mode’s impact on HRQoL are unclear. Several studies have shown that UUI affects emotional functioning more after a caesarean delivery, whereas the impact of SUI on emotional health and HRQoL did not differ according to mode of delivery [5, 7].

A prior publication from the cohort examined in this study showed that SUI was the predominant subtype of postpartum incontinence. At a population level, vaginal delivery was the major risk factor for SUI, followed by reporting SUI during pregnancy [10]. It is of great interest to investigate whether these symptoms actually bother the women, affecting their HRQoL during pregnancy and postpartum. To address this, we used data from the aforementioned large cohort study, which prospectively collected data from early pregnancy up to 1 year postpartum.

This study had three aims: first, to assess bothersome symptoms of PFD during pregnancy and up to 1 year postpartum; second, to study the impact of PFD on HRQoL; and third, to examine whether maternal factors and pregnancy and delivery characteristics affect the prevalence of bothersome UI.

## Materials and Methods

### Study Design and Population

A prospective cohort study (Pelvic Floor in Pregnancy and Childbirth, POPRACT) was conducted in Region Örebro County, Sweden. The full description of the methodology has been published elsewhere [11]. Briefly, all eligible nulliparous women registering for maternity health care in early pregnancy from 1 October 2014 to 1 October 2017 were invited to participate by their midwife. Exclusion criteria were insufficient knowledge of Swedish and a gestational age > 15 weeks + 6 days. Participants completed a web-based questionnaire in early pregnancy, late pregnancy, 8 weeks postpartum, and 12 months postpartum. The questionnaire in early pregnancy collected information on educational level, smoking, and whether urinary leakage had occurred before pregnancy. Data on height and weight were collected at 1 year postpartum. All four questionnaires included questions on PFD [12], pelvic floor bother [4, 13], and HRQoL [13] (Appendix 1). The present study included women who completed the questionnaire on at least one of the four occasions.

### Outcome Measures

We examined three outcomes in this study. For the first aim, we examined several types of bothersome PFD symptoms: UI, SUI, UUI, MUI, FI solid, FI liquid, and vaginal bulging. A bothersome symptom was defined if the woman reported having been bothered “moderately” or “quite a bit.” The questions and answer options are listed in Appendix 1. To study the impact of PFD on HRQoL for the second aim, HRQoL was measured using the Incontinence Impact Questionnaire, Short Form (IIQ-7) [14] (Appendix 2). It consists of seven questions regarding physical activity, travel, social activity, and emotional health. The score ranges from 0 to 100, and the higher the score, the worse the symptoms and the HRQoL. For the third aim, we used bothersome UI measured at 1 year postpartum.

### Exposure Measures

The main exposure variables were time for the first and second aims, and maternal factors and pregnancy and delivery characteristics for the third aim. Self-reported health was dichotomized as either good (“excellent,” “very good,” and “good”) or poor (“fair” and “bad”). Educational level was classified as elementary school, high school, or university. Body mass index (BMI) was calculated from the questionnaire at 1 year postpartum. It was grouped as ≤ 25, 25.1–30, or > 30. Age at delivery was grouped as ≤ 25, 26–30, or > 30 years. Data concerning obstetric variables were extracted from the obstetric record system and from a study-specific delivery protocol including data on perineal tears [15]. Delivery mode was grouped as spontaneous vaginal, vaginal with vacuum extraction, or cesarean section. Perineal tears were categorized into three groups: no laceration or first-degree tears, second-degree tears, or obstetric anal sphincter injury. Birth weight was dichotomized as < 4,000 g and ≥ 4,000 g. UI before pregnancy, bothersome UI in late pregnancy, and bothersome UI at 8 weeks postpartum were examined to see whether they were associated with the risk of bothersome UI 1 year postpartum.

### Statistical Analyses

For the first aim, we calculated the prevalence of bothersome symptoms at each time point. We then tested the difference in the prevalences among all time points using Cochran’s Q test. Although the prevalence was calculated using all available data, Cochran’s Q test was performed among those who completed questionnaires at all time points. For the second aim, we examined the distribution of IIQ-7 scores using prevalence, median, and range. The group reporting any pelvic floor symptoms with impairment in HRQoL (IIQ-7 > 0) was dichotomized into those scoring 0 points on the IIQ-7. We then used Cochran’s Q test to test differences in prevalence overall and made pairwise comparisons of all time points.

For the third aim, the associations of bothersome UI 1 year postpartum with maternal factors and pregnancy and delivery characteristics were evaluated using generalized linear models, estimating risk ratios. Unadjusted and adjusted risk ratios were obtained. Adjustments were made for age and BMI. Any *p* values < 0.05 were considered statistically significant. Data were analyzed using version 16 of Stata/SE (StataCorp LP).

## Results

**Fig. 1:**
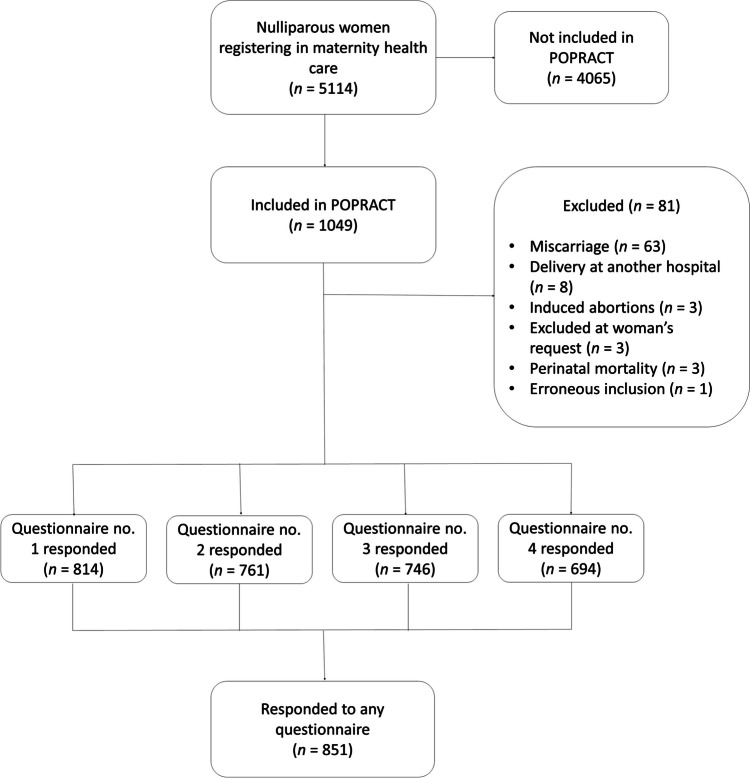
Flowchart illustrating the inclusion of the study sample. *POPRACT* Pelvic Floor in Pregnancy and Childbirth

Of the 1,049 women included in the POPRACT study, 851 completed the questionnaire on at least one of the four occasions (Fig. 1). Most women were < 30 years of age, had a BMI < 30 kg/m^2^, were university educated, and had experienced a vaginal delivery (Table 1). Most women reported good or very good health in early pregnancy.

**Table 1:** Maternal and obstetric baseline characteristics (*N* = 851)

	*n* (%)
Age	
≤ 25 years	158 (18.6)
26–30 years	427 (50.2)
> 30 years	255 (29.9)
Missing	11 (1.3)
BMI	
≤ 25 kg/m^2^	404 (47.5)
25.1–30 kg/m^2^	179 (21.0)
> 30 kg/m^2^	100 (11.8)
Missing	168 (19.7)
Educational level	
< 9 years	14 (1.6)
9–12 years	277 (32.6)
University	521 (61.2)
Missing	39 (4.6)
Smoking	
Yes	20 (2.4)
No	789 (92.7)
Missing	42 (4.9)
Self-reported health in early pregnancy	
Excellent	117 (13.8)
Very good	420 (49.4)
Good	256 (30.1)
Fair	19 (2.2)
Bad	2 (0.2)
Missing	37 (4.4)
UI before pregnancy	
Yes	90 (10.6)
No	761 (89.4)
Missing	0
Delivery mode	
Vaginal	577 (67.8)
Vacuum extraction	135 (15.9)
Cesarean section	128 (15.0)
Missing	11 (1.3)
Perineal tear	
No injury or first degree	262 (30.8)
Second degree	256 (30.1)
Obstetric anal sphincter injury	40 (4.7)
Missing	293 (34.4)
Birth weight	
< 4,000 g	698 (82.0)
≥ 4,000 g	140 (16.5)
Missing	13 (1.5)
Manual perineal protection	
None	29 (3.4)
Fetal head support only	22 (2.6)
Perineal head support only	73 (8.6)
Combined support	363 (42.7)
Unspecified support	160 (18.8)
Missing	204 (24.0)
Episiotomy	
No	515 (60.5)
Yes	50 (5.9)
Missing	286 (33.6)

*BMI* body mass index, *UI* urinary incontinence

The prevalence of bothersome UI increased from 0.7% in early pregnancy to 8.1% at 1 year postpartum. There was also an increasing prevalence from late pregnancy to 1 year postpartum. When UI was divided into subgroups, bothersome SUI was the most common subtype and the prevalence increased over time. The prevalence of bothersome MUI increased 1 year postpartum compared with early and late pregnancy. Very few women reported bothersome UUI and FI at all time points (Table 2).

**Table 2:** The prevalence of bothersome pelvic floor symptoms during pregnancy and postpartum: reporting moderate to great bother was considered to indicate a bothersome symptom

	Early pregnancy, *n* (%)	Late pregnancy, *n* (%)	8 weeks postpartum, *n* (%)	1 year postpartum, *n* (%)
Urinary incontinence, overall	6 (0.7)	28 (3.7)*	40 (5.4)*	56 (8.1)*, **
Stress urinary incontinence	1 (0.1)	20 (2.7)*	14 (1.9)*	27 (3.9)*, ***
Urgency urinary incontinence	3 (0.4)	3 (0.4)	7 (0.9)	6 (0.9)
Mixed urinary incontinence	2 (0.2)	3 (0.4)	12 (1.6)*	17 (2.5)*, **
Fecal incontinence, liquid	6 (0.7)	9 (1.2)	15 (2.0)	14 (2.0)
Fecal incontinence, solid	2 (0.3)	3 (0.4)	7 (0.9)	5 (0.7)

*p* values in the footnotes were obtained using Cochran’s Q test

*p < 0.05; reference early pregnancy

**p < 0.05; reference late pregnancy

***p < 0.05; reference 8 weeks postpartum

**Fig. 2:**
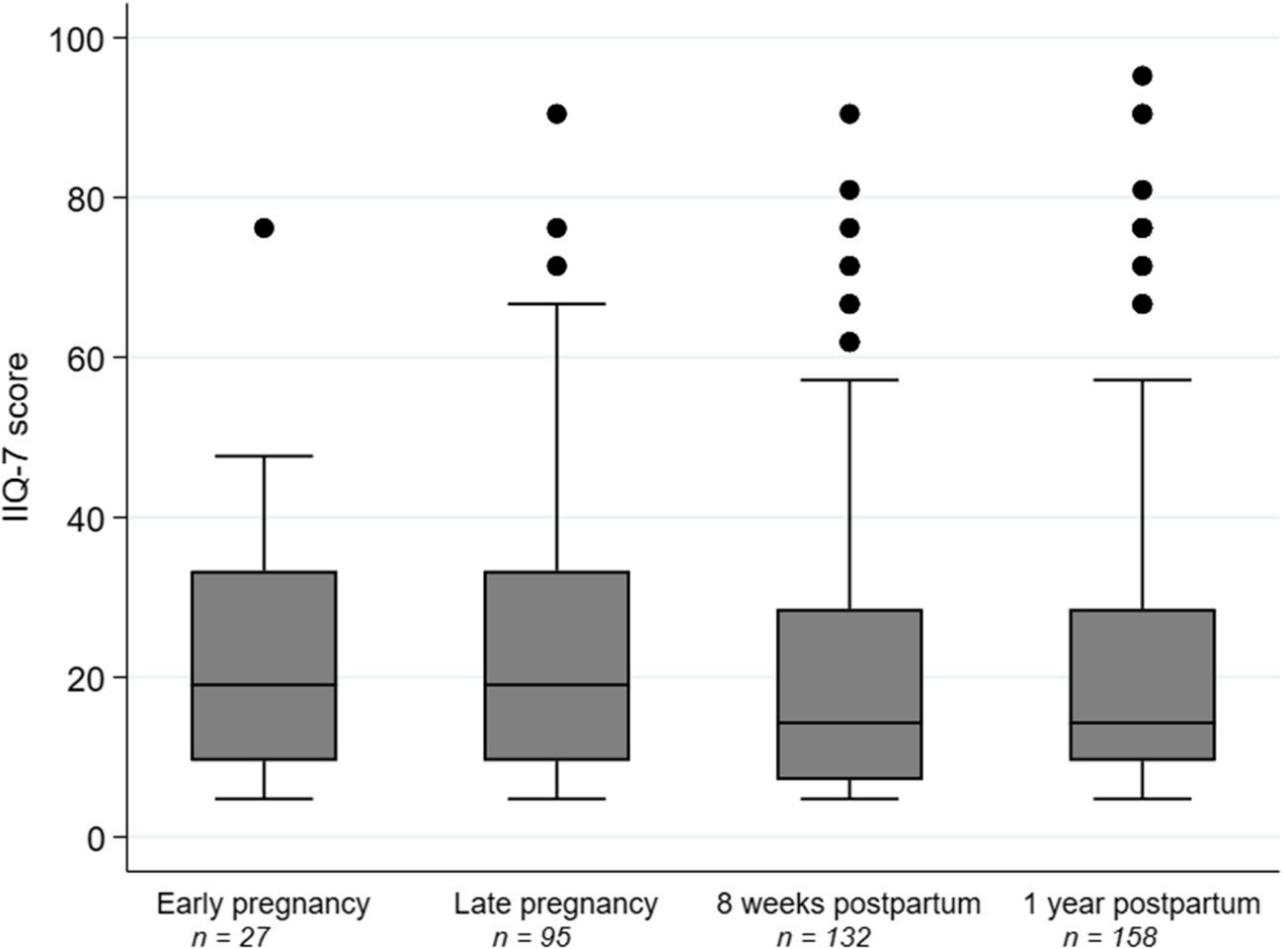
Box-plot of Incontinence Impact Questionnaire, Short Form (IIQ-7) scores during pregnancy and postpartum

The prevalence of impaired HRQoL due to pelvic floor symptoms reported on IIQ-7 is presented in Table 3 and Fig. 2. At 1 year postpartum, 158 women (22.8%) reported any pelvic floor symptoms involving impairment in HRQoL (IIQ-7 score > 0), with a median IIQ-7 score of 14.3. The proportion of women reporting impairment in HRQoL increased from early pregnancy to all other timepoints. There was also an increasing prevalence from late pregnancy to 8 weeks postpartum and 1 year postpartum and from 8 weeks postpartum to 1 year postpartum. Overall, the scores were low, but a few outliers reported high scores. The most common symptom among those with impaired HRQoL 1 year postpartum was SUI, reported by 63 women (9.1%) with a median IIQ-7 score of 19.0 (Table 3).

**Table 3:** The prevalence of impaired health-related quality of life due to pelvic floor symptoms from early pregnancy to 1 year postpartum

	Early pregnancy	Late pregnancy	8 weeks postpartum	1 year postpartum
Total number of participants	814	761	746	694
Any pelvic floor symptom with impairment in HRQoL^a^				
*n* (%)	27 (3.3)	95 (12.5)*	132 (17.7)*, **	158 (22.8)*, **, ***
IIQ-7 median	19.0	19.0	14.3	14.3
Minimum to maximum	4.8–76.2	4.8–90.5	4.8–90.5	4.8–95.2
Stress urinary incontinence				
*n* (%)	4 (0.5)	39 (5.1)	37 (5.0)	63 (9.1)
IIQ-7 median	14.3	14.3	14.3	19.0
Minimum to maximum	4.8–38.1	4.8–71.4	4.8–80.9	4.8–95.2
Urgency urinary incontinence				
*n* (%)	6 (0.7)	4 (0.5)	9 (1.2)	11 (1.9)
IIQ-7 median	14.3	19.0	9.5	28.6
Minimum to maximum	4.8–42.9	4.8–52.4	4.8–38.1	9.5–52.4
Mixed urinary incontinence				
*n* (%)	3 (0.7)	19 (2.5)	20 (2.7)	30 (4.3)
IIQ-7 median	28.6	14.3	28.6	26.2
Minimum to maximum	28.6–42.9	4.8–66.7	4.8–71.4	4.8–90.5
Vaginal bulging				
*n* (%)	3 (0.4)	8 (1.1)	33 (4.4)	35 (5.0)
IIQ-7 median	38.1	16.7	28.6	19.0
Minimum to maximum	14.3–76.2	9.5–71.4	4.8–90.5	4.8–90.5
Fecal incontinence, liquid				
*n* (%)	3 (0.4)	10 (1.3)	20 (2.7)	23 (3.3)
IIQ-7 median	33.3	30.9	19.0	14.3
Minimum to maximum	19.0–47.6	4.8–76.2	4.8–71.4	4.8–47.6
Fecal incontinence, solid				
*n* (%)	1 (0.1)	3 (0.4)	11 (1.5)	7 (1.0)
IIQ-7 median	19.0	33.3	9.5	23.8
Minimum to maximum		28.6–61.9	4.8–47.6	9.5–42.9

IIQ-7 scores are presented as medians and ranges for all types of pelvic floor dysfunction. Participants reporting any impairment are presented in absolute numbers (*n*) and percentages; *p* values in the footnotes were obtained using Cochran’s Q test

*IIQ-7* Incontinence Impact Questionnaire, Short Form

*p < 0.05; reference early pregnancy

**p < 0.05; reference late pregnancy

***p < 0.05; reference 8 weeks postpartum

^a^A woman reporting any pelvic floor symptoms and who had IIQ-7 > 0

Table 4 shows the association of bothersome UI with maternal factors at 1 year postpartum, and the association of bothersome UI with pregnancy and delivery characteristics at 1 year postpartum. Ninety women reported UI before pregnancy, and these women were excluded from the second association. Among the maternal factors studied, UI before pregnancy was associated with bothersome UI 1 year postpartum (adjusted RR 4.36, 95% CI 2.62–7.24). Among the pregnancy- and delivery-related factors studied, bothersome UI in late pregnancy (adjusted RR 4.51, 95% CI 1.43–14.26) and bothersome UI 8 weeks postpartum (adjusted RR 10.17, 95% CI 5.45–18.98) were associated with bothersome UI 1 year postpartum.

**Table 4:** Association of maternal factors with bothersome UI at 1 year postpartum and of pregnancy and delivery characteristics and bothersome UI at 1 year postpartum

	No bothersome UI, *n* (%)	Bothersome UI, *n* (%)	Unadjusted RR (95% CI)	Adjusted RR (95% CI)
Maternal factors				
Self-reported health in early pregnancy				
Good	12 (92.3)	1 (7.7)	–	–
Poor	594 (92.0)	52 (8.0)	0.95 (0.14–6.38)	0.96 (0.14–6.43)
Educational level				
< 9 years	8 (88.9)	1 (11.1)	1.37 (0.20–9.16)	1.39 (0.19–8.76)
9–12 years	200 (92.2)	17 (7.8)	–	–
University	396 (91.9)	35 (8.1)	0.99 (0.57–1.70)	1.10 (0.60–1.99)
Smoking				
No	12 (85.7)	2 (14.3)	–	–
Yes	592 (92.1)	51 (7.9)	1.80 (0.49–6.65)	1.71 (0.45–6.46)
UI before pregnancy				
No	577 (94.0)	37 (6.0)	–	–
Yes	51 (73.9)	18 (26.1)	4.22 (2.55–6.99)*	4.36 (2.62–7.24)*
BMI				
≤ 25	375 (92.8)	29 (7.2)	–	–
25.1–30	163 (91.1)	16 (8.9)	1.25 (0.69–2.23)	–
> 30	90 (90.0)	10 (10.0)	1.39 (0.70–2.76)	–
Age				
≤ 25 years	114 (91.9)	10 (8.1)	–	–
26–30 years	326 (92.4)	27 (7.6)	0.95 (0.47–1.90)	–
> 30 years	198 (91.2)	19 (8.8)	1.09 (0.52–2.26)	–
Pregnancy and delivery characteristics				
Delivery mode				
Vaginal	389 (92.6)	31 (7.4)	–	–
Vacuum extraction	96 (95.0)	5 (5.0)	0.63 (0.25–1.59)	0.69 (0.27–1.72)
Cesarean section	92 (98.9)	1 (1.1)	0.14 (0.02–1.03)	0.14 (0.021–1.05)
Perineal tear				
No injury or first degree	173 (91.5)	16 (8.5)	–	–
Second degree	182 (92.2)	14 (7.1)	0.82 (0.041–1.61)	0.88 (0.44–1.77)
Obstetric anal sphincter injuries	30 (96.8)	1 (3.2)	0.37 (0.05–2.69)	0.42 (0.06–3.04)
Birth weight				
< 4,000 g	469 (81.4)	31 (18.6)	–	–
≥ 4,000 g	107 (83.8)	6 (16.2)	0.85 (0.36–1.98)	0.84 (0.36–1.96)
Bothersome UI in late pregnancy				
No	526 (98.3)	31 (1.7)	–	–
Yes	9 (91.2)	3 (8.8)	4.42 (1.57–12.46)*	4.51 (1.43–14.26)*
Bothersome UI 8 weeks postpartum				
No	533 (97.4)	24 (2.6)	–	–
Yes	14 (70.6)	10 (29.4)	9.04 (4.89–16.71)*	10.17 (5.45–18.98)*, ^a^

Adjusted model adjusted for age and BMI

In the pregnancy and delivery characteristics, women with UI before pregnancy were excluded from the analysis

*BMI* body mass index, *UI* urinary incontinence

^*^p < 0.05

^a^Age was grouped into ≤ 30 and > 30 years because the model did not have convergence with three age categories

## Discussion

In this large prospective cohort study of primiparous women, we evaluated bothersome PFD and its impact on HRQoL during pregnancy and up to 1 year postpartum. We found that the prevalence of bothersome UI increased significantly during pregnancy and up to 1 year postpartum, and this increase was seen in the groups reporting bothersome SUI and MUI. Very few women reported bothersome UUI and FI at any time point. Risk factors for bothersome UI 1 year postpartum were UI before pregnancy and bothersome UI in late pregnancy and at 8 weeks postpartum. Almost every fourth woman reported pelvic floor symptoms with an impact on HRQoL 1 year postpartum, but the median scores in IIQ-7 were low and most women did not report any effect on HRQoL at all.

To the best of our knowledge, only one previous study has examined bothersome urinary symptoms during pregnancy and up to 1 year postpartum [8]. In contrast to our finding that bothersome UI increased during pregnancy and up to 1 year after childbirth, Van Brummen et al. found that the prevalence of bothersome LUTS was highest at week 36 of gestation and declined 1 year after pregnancy, suggesting that it might be part of normal pregnancy [8]. Palmieri et al. also found that bothersome PFD was more prevalent in pregnancy than postpartum, but they only followed their cohort up to 6 weeks postpartum [16]. Van Brummen et al. found that bothersome SUI in early pregnancy and greater maternal age were predictive of bothersome SUI 1 year after childbirth [8]. In the present study, the small number of women reporting bothersome UI in early pregnancy also reported UI before pregnancy, so we used that as a predictor of bothersome UI 1 year postpartum. The finding that bothersome UI in late pregnancy and 8 weeks postpartum were predictive of bothersome UI 1 year postpartum is similar to the results of previous studies [17, 18]. However, both these previous studies focused on UI in general and not specifically on bothersome UI. Foldspang et al. reported that the highest risk of postpartum UI was found among women complaining of UI during pregnancy, which manifested as a crucial, independent precursor [18].

In the present study, few women reported bothersome FI at all time points. When comparing our lower FI prevalence figures with those reported in previous studies of FI not applying cut-offs based on bother [19, 20], it is clear that only a fraction of women with FI are bothered by it.

Few studies have evaluated the impact of PFD on HRQoL after pregnancy and childbirth [5, 7–9]. A minority of women with UI reported an impact on HRQoL in our study. Previous studies have reported various results. Dolan et al. reported that, during pregnancy, most women with UI experience minimal impact on HRQoL [9]. In contrast, in other studies UI had a significant impact on HRQoL during pregnancy [21, 22]. In a general population, Coyne et al. reported that women with UUI and MUI had significantly worse HRQoL than did women with SUI [23]. This is similar to our finding that women with MUI and UUI had the highest median IIQ-7 score 1 year postpartum; however, because the groups were small, it was not possible to conduct proper statistical evaluations. Even though the impairment in HRQoL in our total sample is low, it should be noted that some outliers have very high scores. These few women have more extensive limitations in their daily activities. When looking at the detailed IIQ-7 answers 1 year postpartum (Appendix 3), it seems as though physical activity is the domain with the highest score. That is probably because SUI, the most common symptom, is closely associated with physical activity.

The association between delivery mode and bothersome UI 1 year postpartum did not reach statistical significance. However, this was not the main objective of the study. A larger study sample and a longer follow-up time would be required to establish the effect of delivery mode on bothersome UI symptoms, as well as other bothersome PFD. Van Brummen et al. reported that women had more bothersome UUI after a cesarean delivery and more bothersome SUI after a vaginal delivery, but neither effect was statistically significant [8]. As other previous studies of postpartum UI have not focused on bothersome UI, we cannot entirely compare our results with theirs [5, 7, 17, 18]. It would have been interesting to include UI subgroups as well as bothersome FI in the association analysis, but the groups were too small.

### Strengths and limitations

A strength of this study is the prospective data collection from early pregnancy to 1 year postpartum. As the study sample comprised only primiparous women, the results were not influenced by previous pregnancy or childbirth. In general, the likelihood of recall bias is low in studies of prospective design. We consider it a strength to focus on bothersome PFD and impairment in HRQoL, because these, rather than symptom severity, are the forces that drive women to seek treatment [6]. Our questionnaire was a compilation of questions derived from various validated questionnaires. Compiling questions from several questionnaires may influence the validated characteristics of the questions [24]. Unfortunately, the questionnaire did not include any questions about bothersome vaginal bulging, limiting our understanding of how this symptom may bother women. The IIQ-7 is a standardized validated questionnaire for UI. In this study, we used a modified Swedish version asking about several pelvic floor symptoms, but the answer categories were not divided into different symptoms as in the PFIQ-7. Therefore, we cannot tell what PFD symptoms may have affected the HRQoL. Another limitation of IIQ-7 is that the questionnaire is constructed for condition-specific HRQoL among women with UI in clinical practice and is not validated for young women who have recently given birth. This has probably introduced floor effects, i.e., that most respondents score 0 points on the IIQ-7, making it difficult to manage and analyze the data. Finally, our definitions of bothersome PFD are not standardized or based on any core outcome set, as no core outcome set is available in this field [25]. There is an urgent need to define a core outcome set for PFD to make the results of different studies comparable.

Our findings indicate a low overall prevalence of bothersome UI during pregnancy and up to 1 year after first childbirth and that most women did not report PFD with impairment in HRQoL. This is a reassuring message for pregnant women. However, the minority of women actually suffering from bothersome UI and other PFD must be identified and adequately counseled in postnatal care. Studies have shown that women with SUI during first pregnancy or 3 months after first delivery are at a particularly high risk of long-lasting symptoms 5–15 years after childbirth [26, 27]. Altogether, this further supports the introduction of preventive measures, such as pelvic floor muscle training during pregnancy [28].

## Conclusions

The prevalence of bothersome UI increased during pregnancy and postpartum, suggesting that both pregnancy and childbirth might contribute to these symptoms. Most women were not bothered by UI or any other PFD during pregnancy and up to 1 year after their first childbirth, and did not report any pelvic floor symptoms causing impairment in HRQoL. However, there is a need to emphasize that a small number of women reported more extensive limitations in their daily living. Bothersome UI in late pregnancy and 8 weeks postpartum were predictive of bothersome UI 1 year postpartum. These results indicate the importance of identifying these women during pregnancy and at the postnatal check-up in order to provide appropriate counseling and treatment to reduce their problems.

## Data Availability

The data that support the findings of this study are available from the corresponding author upon reasonable request.

## Appendix 1

Table 5 shows questions on urinary leakage, fecal incontinence, and symptoms of prolapse.

**Table 5:** Questions on urinary leakage, fecal incontinence, and symptoms of prolapse

Outcome and corresponding question	Answer options	Definition of outcome
Urinary leakage overall		
Q1: Do you occasionally experience urinary leakage? [12]	Yes	
	No	
If Yes, how much does it bother you? [13]	Not at all	Answering “Moderately” or “Quite a bit” Q1 → Bothersome UI overall
	Somewhat	
	Moderately	
	Quite a bit	
UUI		
Q2: Do you occasionally experience a sudden need to urinate and then have difficulty reaching the toilet in time? [4]	Yes, often	Answering “Yes, often” or “Sometimes” on Q2 and “Infrequently” or “No, never” on Q3 → UUI; UUI + Q1 = moderately or quite a bit → Bothersome UUI
	Sometimes	
	Infrequently	
	No, never	
SUI		
Q3: Do you leak urine when coughing, sneezing, lifting, or during physical activities? [4]	Yes, often	“Yes, often” or “Sometimes” on Q3 and “Infrequently” or “No, never” on Q2 → SUI; SUI + Q1 = moderately or quite a bit → Bothersome SUI
	Sometimes	
	Infrequently	
	No, never	
MUI		
Questions #2 and #3		“Yes, often” or “Sometimes” to both question #2 and #3 → MUI; MUI + Q1 = moderately or quite a bit → Bothersome MUI
FI, liquid		
Q4: Do you usually lose stool beyond your control if your stool is loose? [13]	Yes	
	No	
If Yes, how much does it bother you? [13]	Not at all	Answering “Moderately” or “Quite a bit” Q4 → Bothersome FI liquid
	Somewhat	
	Moderately	
	Quite a bit	
FI, solid		
Q5: Do you usually lose stool beyond your control if your stool is well formed? [13]	Yes	
	No	
If Yes, how much does it bother you? [13]	Not at all	Answering “Moderately” or “Quite a bit” Q5 → Bothersome FI
	Somewhat	
	Moderately	
	Quite a bit	
Symptom of prolapse		
Q6: Do you have a sensation of tissue protruding from your vagina (vaginal bulge)? [4]	Yes, often	Answering “Yes, often” or “Sometimes” Q6
	Sometimes	
	Infrequently	
	No, never	

*UI* urinary incontinence, *UUI* urge urinary incontinence, *SUI* stress urinary incontinence, *MUI* mixed urinary incontinence, *FI* fecal incontinence

## Appendix 2

The questions of the IIQ-7 are shown below [14].

Do any symptoms from the pelvic floor bother you? (urinary leakage, gas/stool leakage, prolapse)

- Yes
- No

If yes, to what extent do those symptoms affect your:

- Ability to do household chores (cooking, housecleaning, laundry)?
  - Not at all
  - Slightly
  - Moderately
  - Quite a bit
- Ability to do physical activity such as walking, swimming, or other exercise?
  - Not at all
  - Slightly
  - Moderately
  - Quite a bit
- Entertainment activities such as going to a movies or concert?
  - Not at all
  - Slightly
  - Moderately
  - Quite a bit
- Ability to travel by car or bus for a distance greater than 30 min away from home?
  - Not at all
  - Slightly
  - Moderately
  - Quite a bit
- Participation in social activities outside your home?
  - Not at all
  - Slightly
  - Moderately
  - Quite a bit
- Emotional health (nervousness, depression, etc.)?
  - Not at all
  - Slightly
  - Moderately
  - Quite a bit
- Feeling frustrated?
  - Not at all
  - Slightly
  - Moderately
  - Quite a bit

## Appendix 3

The answers given to the IIQ-7 at 1 year postpartum are shown in Table 6.

**Table 6:** Incontinence Impact Questionnaire, Short Form answers at 1 year postpartum

	1 year postpartum *n* (%)
Do any symptoms from the pelvic floor bother you? (urinary leakage, gas/stool leakage, prolapse)	
Total	694
Yes	200 (28.8)
No	494 (71.2)
Missing	0
If yes, to what extent do those symptom affect your	
Ability to do household chores (cooking, housecleaning, laundry)?	
Total	200
Not at all	169 (84.5)
Slightly	18 (9.0)
Moderately	5 (2.5)
Quite a bit	7 (3.5)
Missing	1 (0.5)
Ability to do physical activity such as walking, swimming, or other exercise?	
Total	200
Not at all	59 (29.5)
Slightly	88 (44.0)
Moderately	38 (19.0)
Quite a bit	13 (6.5)
Missing	2 (1.0)
Entertainment activities such as going to a movies or concert?	
Total	200
Not at all	151 (75.5)
Slightly	29 (14.4)
Moderately	13 (6.5)
Quite a bit	6 (3.0)
Missing	1 (1.0)
Ability to travel by car or bus for a distance greater than 30 min away from home?	
Total	200
Not at all	153 (76.5)
Slightly	30 (15.0)
Moderately	7 (3.5)
Quite a bit	8 (4.0)
Missing	2 (1.0)
Participation in social activities outside your home?	
Total	200
Not at all	135 (67.5)
Slightly	49 (24.5)
Moderately	6 (3.0)
Quite a bit	9 (4.5)
Missing	1 (0.5)
Emotional health (nervousness, depression, etc.)?	
Total	200
Not at all	104 (52.0)
Slightly	67 (33.5)
Moderately	19 (9.5)
Quite a bit	8 (4.0)
Missing	2 (1.0)
Feeling frustrated?	
Total	200
Not at all	100 (50.0)
Slightly	67 (33.5)
Moderately	21 (10.5)
Quite a bit	11 (5.5)
Missing	1 (0.5)

## Abbreviations

BMI: Body mass index
FI: Fecal incontinence
HRQoL: Health-related quality of life
IIQ-7: Incontinence Impact Questionnaire, Short Form
LUTS: Lower urinary tract symptoms
MUI: Mixed urinary incontinence
PFD: Pelvic floor dysfunction
PFIQ-7: Pelvic Floor Impact Questionnaire, Short Form
POP: Pelvic organ prolapse
POPRACT: Pelvic Floor in Pregnancy and Childbirth
SUI: Stress urinary incontinence
UI: Urinary incontinence
UUI: Urgency urinary incontinence
